# Three-Dimensional Structure of Dendritic Spines Revealed by Volume Electron Microscopy Techniques

**DOI:** 10.3389/fnana.2021.627368

**Published:** 2021-05-31

**Authors:** Laxmi Kumar Parajuli, Masato Koike

**Affiliations:** ^1^Department of Cell Biology and Neuroscience, Juntendo University Graduate School of Medicine, Tokyo, Japan; ^2^Advanced Research Institute for Health Science, Juntendo University, Tokyo, Japan

**Keywords:** dendritic spine, synapse, electron microscopy, 3D reconstruction, immunogold

## Abstract

Electron microscopy (EM)-based synaptology is a fundamental discipline for achieving a complex wiring diagram of the brain. A quantitative understanding of synaptic ultrastructure also serves as a basis to estimate the relative magnitude of synaptic transmission across individual circuits in the brain. Although conventional light microscopic techniques have substantially contributed to our ever-increasing understanding of the morphological characteristics of the putative synaptic junctions, EM is the gold standard for systematic visualization of the synaptic morphology. Furthermore, a complete three-dimensional reconstruction of an individual synaptic profile is required for the precise quantitation of different parameters that shape synaptic transmission. While volumetric imaging of synapses can be routinely obtained from the transmission EM (TEM) imaging of ultrathin sections, it requires an unimaginable amount of effort and time to reconstruct very long segments of dendrites and their spines from the serial section TEM images. The challenges of low throughput EM imaging have been addressed to an appreciable degree by the development of automated EM imaging tools that allow imaging and reconstruction of dendritic segments in a realistic time frame. Here, we review studies that have been instrumental in determining the three-dimensional ultrastructure of synapses. With a particular focus on dendritic spine synapses in the rodent brain, we discuss various key studies that have highlighted the structural diversity of spines, the principles of their organization in the dendrites, their presynaptic wiring patterns, and their activity-dependent structural remodeling.

## Introduction

Understanding the precise ultrastructure of synapses is essential to unravel the intricate neuronal circuitry and physiological functions of the brain. In many regions of the brain, excitatory synaptic contacts are formed on tiny dendritic protrusions known as dendritic spines (Harris and Weinberg, 2012; Frotscher et al., 2014; Parajuli et al., 2017; Parajuli, 2018). Owing to their role in mediating neuronal excitability and biochemical signaling, dendritic spines have been intensely studied using multiple experimental approaches, including biochemical, electrophysiological, molecular biological, and imaging techniques. The peculiar bulbous morphology of individual dendritic spines can be readily identified in light microscopy (LM) preparations (Okabe, 2020). In recent years, super-resolution microscopes that break the diffraction barrier of conventional LM techniques are becoming increasingly popular and powerful tools to image the surface geometry of dendritic spines in live preparation (Chereau et al., 2015; Kashiwagi et al., 2019). However, despite the technical developments in LM imaging tools; volume electron microscopy (EM) techniques are indispensable for the visualization of high-resolution three-dimensional structures and the precise quantification of various morphological parameters that govern synaptic transmission. Here, we review the subcellular structure of excitatory synapses, with a particular focus on dendritic spine structure obtained from volume EM studies.

Much of our knowledge of the three-dimensional structure of dendritic spines has been obtained from serial-section transmission EM (TEM) studies, in which resin-embedded brain sections are sliced into ribbons of serial ultrathin sections, typically 40–80 nm thick (Wilson et al., 1983; Harris and Stevens, 1988, 1989; Harris et al., 1992; Ichikawa et al., 2002, 2016; Stewart et al., 2005, 2010; Medvedev et al., 2010, 2014; Mishchenko et al., 2010). Once serial images are acquired, they are aligned, and the same neuronal profile is traced in each section to render a three-dimensional shape. Serial section TEM studies can be performed in any basic EM facility that is equipped with a regular conventional TEM. Specialized, advanced equipment is not necessary for three-dimensional studies using serial section TEM.

**Figure 1:**
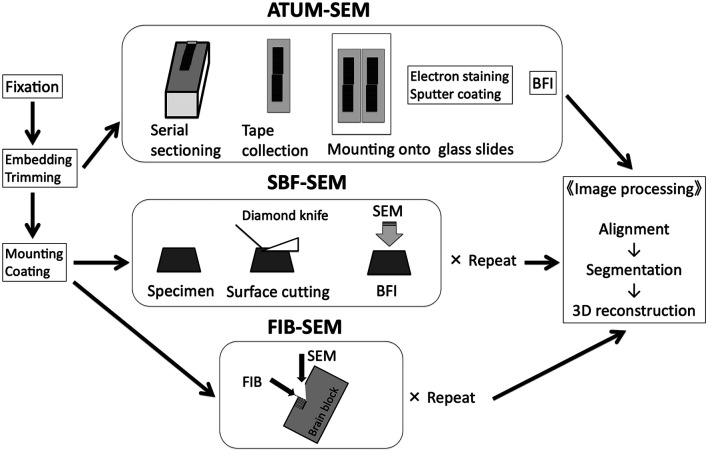
Basic workflow of three different SEM-based volume imaging methods. ATUM-SEM, automated tape collecting ultramicrotome scanning electron microscopy; BFI, block-face imaging; FIB-SEM, focused ion beam scanning electron microscopy; SBF-SEM, serial block-face scanning electron microscopy. Modified from Koike (2018) with permission. In our lab, we use FIB-SEM to typically image a cube of 10 μm × 10 μm × 10 μm for three-dimensional reconstruction of dendritic shafts and spines.

A principal advantage of serial ultrathin sections is that once the sections are obtained, they can be safely stored in a grid case for several years for later use and the same grid (or even the same neuronal profile) can be reimaged multiple times. In spite of this, the biggest bottleneck in the use of serial section TEM is that a great deal of skilled labor is required to cut ribbons of serial sections that can break, be missed, or distorted during sectioning and sample collection on the metal grids. Typically, reconstructions are performed using 20–30 serial sections collected on a single grid (Nicholson et al., 2006; Parajuli et al., 2010; Aziz et al., 2014). As a result, the limited neuropil volume permits full reconstruction of dendritic spines, but the imaged volume is not large enough to reconstruct an appreciable length of a dendritic shaft. The length of the reconstructed dendrites can be increased by collecting 100–200 serial sections in multiple grids (Ostroff et al., 2002; Harris et al., 2006; Kulik et al., 2019). However, this is technically highly demanding and only a few laboratories have this capacity on a routine basis. Heroic studies, such as the complete reconstruction of 302 neurons in *Caenorhabditis* *elegans* (White et al., 1986), reconstruction of a considerable length of a dendritic segment in the thalamus (Hamos et al., 1987) have been performed by extensive imaging of more than a thousand serial sections. Unarguably, these studies are tremendously labor-intensive, requiring several years of work. The recent development of custom-built TEM camera array (TEMCA) has greatly enhanced the image acquisition speed and the large area can be imaged by this method. In combination with *in vivo* physiology, TEMCA has been used to study the anatomical features in neurons that share similar functional attributes (Bock et al., 2011; Briggman et al., 2011; Lee et al., 2016). Despite the significant development, TEM-based volume imaging approaches are generally of limited use because of the significant amount of human labor needed for sample preparation, ultrathin sectioning, and image alignment after image acquisition. Thus, in the last couple of decades, there has been a pressing need for the development of EM technologies that can acquire unprecedented neuronal volumes with minimal human intervention. The addition of cutting-edge techniques, such as focused ion beam scanning electron microscopy (FIB-SEM; Knott et al., 2008), serial block-face scanning electron microscopy (SBF-SEM; Denk and Horstmann, 2004) to the EM toolkit highlights some of the recent advances in three-dimensional ultrastructural studies (Figure 1). These technical developments have greatly expanded our capacity to reconstruct several dozen micrometers of a dendrite with an effort that is simply incomparable to that needed for the manual sectioning and imaging of serial sections using conventional TEM methods.

**Figure 2:**
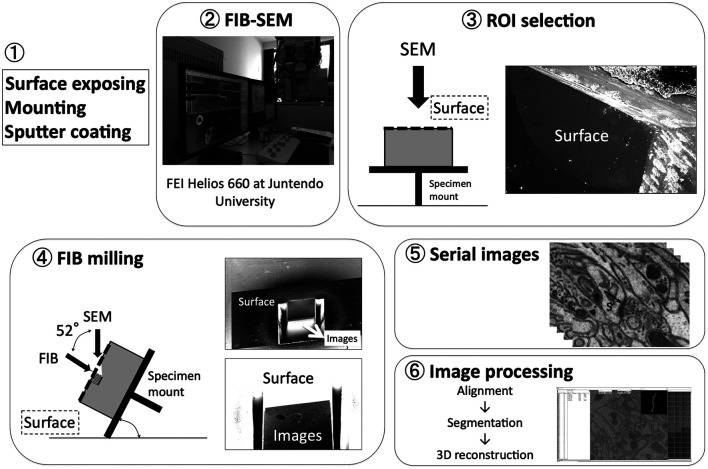
Basic workflow of FIB-SEM. Modified from Koike (2018) with permission. A spine (s) can be clearly visualized with its postsynaptic density (PSD; indicated by black arrow).

Although each imaging tool has its own merits and limitations, the primary difference between the TEM and SEM methods is that the transmitted electrons are used for image formation in the TEM method, and secondary and/or backscattered electrons are used in the case of SEM. Thus, TEM methods are only capable of imaging thin sections. However, SEM can be applied both for thin sections and blocks. TEM images have a higher spatial resolution in the x and y axes compared with SEM methods. In contrast, a FIB-SEM has higher resolution in the z-axis compared with other three-dimensional EM techniques. Slices as thin as 7–10 nm can be sequentially milled from a specimen block face using the gallium ion beams in the FIB-SEM imaging method (Figure 2). As such, FIB-SEM has the advantage over other techniques to visualize fine subcellular structures, such as postsynaptic density (PSD), synaptic vesicles, and intracellular organelles, such as endoplasmic reticulum and mitochondria (Parajuli et al., 2020a). However, array tomography and SBF-SEM have a wider field of view and are more appropriate for studying synaptic connectivity in local neuronal networks. Thus, the choice of imaging method should be decided based on the technical requirement of the research question being asked.

## Three-Dimensional Morphology and The Presynaptic Contacts of Dendritic Spines

**Figure 3:**
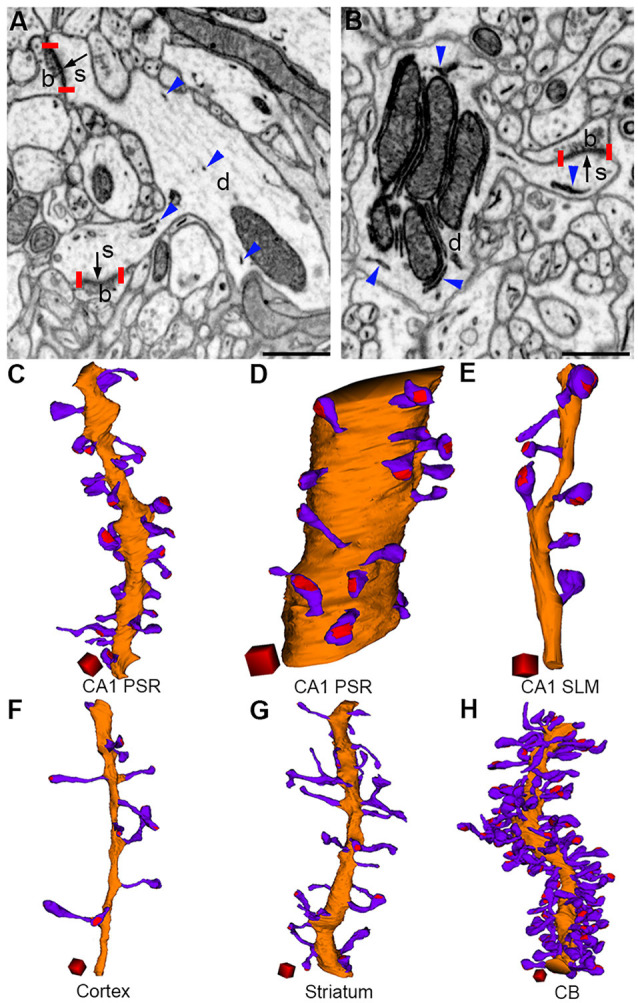
Morphological diversity of dendritic spines in the brain (adapted from Parajuli et al., 2020a). Presynaptic boutons (b), dendrites (d), and spines (s) can be identified in FIB-SEM images from the cerebral cortex **(A)** and cerebellum **(B)**. PSDs (bordered by red vertical bars) are indicated by black arrows and ER are indicated by blue arrowheads. Three-dimensional reconstructions of dendrite (orange), spines (violet), and PSDs (red) from the CA1 proximal stratum radiatum (oblique dendrite, **C**), CA1 proximal stratum radiatum (large-caliber dendrite, **D**), CA1 stratum lacunosum moleculare **(E)**, cortex **(F)**, striatum **(G)**, and cerebellum **(H)**. Scale bars: 500 nm **(A,B)**. Scale cubes: 0.5 μm on each side **(C–H)**.

A dendritic spine typically possesses a spherical head that connects to the dendritic shaft *via* a narrow spine neck (Figure 3). Although the gross anatomical features of spines in different brain regions may appear generally similar, considerable differences can be noticed among them when they are analyzed at the ultrastructural level. We recently performed a quantitative comparison of dendritic spines from the hippocampal CA1, the somatosensory cortex, striatum, and cerebellum. We found significant differences in the dimensions of the spine head and spine neck, and in the density of spines on dendrites among the brain regions (Parajuli et al., 2020a; Figure 3). Despite large differences in spine dimensions, the ratio of the PSD area to neck length was not significantly different between spines in the CA1 and cerebellum. In layered cortical regions (i.e., in the hippocampus, cortex, and cerebellum), dendritic spines were organized in a structured pattern along the dendrite such that the PSD area density (expressed as the PSD area per unit length of the dendrite) positively scaled with dendrite diameter, suggesting that there may be a common principle of spine placement along the dendrite in different neurons.

Based on the relative dimensions of the spine head and spine neck, dendritic spines are generally classified into thin, stubby, and mushroom types (Bourne and Harris, 2008). However, this classification is not exhaustive because there is no clear demarcation in the structural features of spines between these three categories (Kashiwagi et al., 2019). Moreover, there are no well-accepted criteria or molecular markers that differentiate spine head and spine neck. This type of spine classification can also not be applied to brain regions where atypical spine morphology exists. For example, thorny excrescence spines in the CA3 region of the hippocampus are extremely large (Wilke et al., 2013) with profuse branching of the spine heads. The cytosol of these spines contains numerous mitochondria, which is generally excluded from typical dendritic spines in the brain (Parajuli et al., 2020a). Similarly, a recent volume EM study in the inferior colliculus of the barn owl reported doughnut-shaped spines with a hole in the center, without any obvious head and neck like features (Sanculi et al., 2020). Recently, by three-dimensional reconstruction of FIB-SEM images, we revealed atypical U-shaped dendritic protrusions from dendrites of neurons in the interpeduncular nucleus (Parajuli et al., 2020b). Although in single section EM images, some of these protrusions appear to have oval head-like structures that bulge out from the narrow, constricted neck-like structure (Lenn, 1976), clear head and neck like structures are not apparent in the three-dimensional reconstructions. These attributes of dendritic spines highlight a huge diversity in their morphological architecture, which is perhaps necessitated by their functional requirement to perform a vast repertoire of neuronal computations.

Volume EM studies have also markedly enhanced our understanding of the presynaptic connectivity pattern of spines. Three-dimensional reconstruction of neuropil in the cerebellum, cortex, CA1 proximal stratum radiatum, and CA1 stratum laconosum moleculare of the hippocampus have shown that an axon can make synaptic contacts with more than one spine of the same neuron (Harris and Stevens, 1988; Bartol et al., 2015; Kasthuri et al., 2015; Bloss et al., 2018; Parajuli et al., 2020a). The findings in the cerebellum are particularly worth highlighting because it challenges the conventional view that a given parallel fiber forms one synaptic contact with a given Purkinje cell. Serial section EM studies revealed that about 20–25% of the spines had a presynaptic partner that made more than one synaptic contact with the given Purkinje cell neuron (Harris and Stevens, 1988; Parajuli et al., 2020a). A parallel fiber was seen to contact as many as four different spines on a dendrite of a Purkinje cell (Parajuli et al., 2020a). Since the reconstruction volume was limited in these studies, the proportion of spines that share presynaptic axonal varicosities is likely to be considerably higher than what was thought previously. As both climbing fibers and parallel fibers contact spines at multiple locations on the same dendrite, presynaptic action potential invasion can yield robust excitation in Purkinje cell neurons.

A spine generally receives a synaptic input from a single presynaptic varicosity. However, in some brain regions, large spines can receive more than one synaptic input. Approximately 10% of vesicular glutamate transporter 2-positive terminals in the cortex receive an additional synaptic input from GABAergic neurons (Kubota et al., 2007). Although the frequency of GABAergic synaptic contacts varies depending on the cortical layers, double innervated spines were observed in all the layers of the cortex. Perhaps the recently described toric spine represents a prominent example of multiple synaptic inputs that converge onto a single spine. A single toric spine receives synaptic inputs from as many as 11 different axons (Sanculi et al., 2020). This connectivity design is different from that of mossy fiber inputs onto CA3 thorny excrescence spines, where multiple synaptic inputs (as many as 37 synaptic contacts) are made onto one spine by a single presynaptic bouton (Chicurel and Harris, 1992). Thus, the connectivity design can vary depending on the brain region.

## Dendritic Spine Morphology Under Pathological Conditions and Aging

Alteration of dendritic spine density in various neurological conditions was realized quite early on from the panoply of LM studies of Golgi-stained sections (Penzes et al., 2011). Although spine protrusions can be realized fairly accurately in LM preparations (Okabe, 2020), quantification of spine dimensions is not reliable by LM because the head width and neck diameter of small spines are often below the resolution threshold of conventional LM techniques. Thus, fine-scale volume EM studies have been undertaken to quantitatively explore morphological changes in different neurological conditions. Three-dimensional EM studies indicate that the development and maturity of dendritic spines are compromised in disease conditions. FIB-SEM studies show higher spine density and a higher frequency of immature spines in autism spectrum disorder (Sato and Okabe, 2019), and in Parkinson’s disease (Parajuli et al., 2020c). A similar observation was also made using SBF-SEM in fragile X syndrome (Jawaid et al., 2018), and in schizophrenia (Nakao et al., 2017). Together, these studies indicate that in pathological conditions, an increase in the total excitatory drive in the dendrites resulting from an increase in the number of spines is compensated for by a decrease in spine head volume. As a corollary to this, a TEM study showed that homeostatic scaling may also be achieved by an increase in the number of synaptic contact sites in response to the decrease in spine density, as seen in mesial temporal lobe epilepsy (Witcher et al., 2010). However, the homeostatic scaling of synaptic strength that occurs to restore neuronal excitation may not be a general feature for all neurological diseases because either no change in spine density or size (Dominguez-Alvaro et al., 2018), or only a slight decrease in the frequency of spine synapses was revealed in FIB-SEM studies performed in brain samples obtained from Alzheimer’s disease patients (Dominguez-Alvaro et al., 2019). Similarly, a TEM study showed that compared with controls, a decrease in the number and volume of thorns was observed in thorny-excrescence synapses in the Ts65Dn mouse model of Down’s syndrome (Popov et al., 2011).

Homeostatic scaling of synaptic strength also occurs with aging. Dense volume reconstruction of serial section FIB-SEM images in layer 1 of the somatosensory cortex shows lower spine density and higher synapse size in 24-month-old mice compared to that in the 4-month-old mice (Cali et al., 2018). Similarly, we recently used FIB-SEM to compare the spine morphology of striatal neurons in control and A53T bacterial artificial chromosome (BAC) human α-synuclein (A53T-BAC-SNCA) transgenic Parkinson’s mice at 1, 3, 6, and 22 months of age. We revealed that the average spine head volume increases but the spine density gradually decreases with age in wild-type mice (Parajuli et al., 2020c). However, this form of a negative relationship between spine density and head volume was not seen in the mutant mice, indicating altered synaptic homeostasis in neurological conditions.

## Three-Dimensional Distribution of Molecules in Dendritic Spines

Automated volume EM is a powerful technique that simultaneously generates structural and molecular information. The distribution pattern and the number and density of molecules localized at synapses have a profound impact on neuronal excitation and downstream signaling. Most volume imaging studies of molecular localization in dendritic spines have been limited to using ultrathin sections prepared by the pre-embedding immunogold labeling method. The principal advantage of this technique is that it is fairly sensitive for the detection of molecules localized at non-synaptic sites (Parajuli et al., 2010, 2012). However, pre-embedding EM is of limited use in detecting molecules localized in the PSD because of poor antibody penetration into the dense protein scaffolds in the PSD (Masugi-Tokita and Shigemoto, 2007). This problem can be circumvented with a post-embedding immunogold labeling method where immunolabeling is performed on ultrathin sections embedded in hydrophilic resins, such as LR white and Lowicryl.

Volume EM techniques, such as FIB-SEM, have been successfully used to reconstruct neuronal profiles that are labeled by immunoperoxidase staining method (Bosch et al., 2015). However, the diffusible nature of peroxidase does not allow the quantitative analysis of the number and density of molecules in different neuronal compartments. FIB-SEM still has a limited use for the three-dimensional reconstruction of pre-embedding immunogold-labeled specimens. This is mainly because it is difficult to achieve deep antibody penetration into the tissue while maintaining structural integrity. In the future, the use of ultrasmall gold particles (0.8 nm, Aurion) and nanobodies may be useful to increase the labeling efficiency (Van de Plas and Leunissen, 1993; Fang et al., 2018).

The reliability of molecular distribution data relies entirely on the specificity of the antibody used for immunolabeling. Thus, first and foremost, the specificity of the antibody should be assessed using appropriate controls. A single protein band on a Western blot and the absence of staining in sections by the omission of the primary antibody are widely used to document the specificity of an antibody. Ideally, no staining in tissue sections prepared from a knock-out mouse of the molecule under investigation should be confirmed. Moreover, in the situation where a specific antibody against the endogenous protein is not available, genetic engineering tools could be used to insert molecular tags that can serve as epitopes for antibody binding (Mikuni et al., 2016). Genetically encoded EM probes, such as APEX2 (Ascorbate Peroxidase 2) and miniSOG (mini Singlet Oxygen Generator) can also serve as alternative tools to study subcellular localization (Shu et al., 2011; Lam et al., 2015). However, compared with antibody-based immunolabeling methods, where the particulate nature of immunogold permits the quantification of the number and density of molecules, a particular limitation of genetically encoded EM probes is that the diffusible reaction product that is generated to visualize the localization site of the molecule precludes quantitative localization. Although, a gold nanoparticle-based genetically-encoded tag has been recently reported to determine the precise localization of molecules in the mammalian cell lines (Jiang et al., 2020), further methodological refinements may be necessary before it can be used in brain slices.

## Retrospective EM Studies of Dendritic Spines

Retrospective EM is the gold standard method to examine structural changes that accompany various physiological processes *in vivo*. Pioneering studies from Svoboda lab have elegantly combined two-photon *in vivo* imaging with the serial section EM to answer fundamental questions about synapse formation and elimination. *In vivo* imaging of mice that express fluorescent proteins in a subset of neurons have revealed that dendritic shafts are highly stable structures, but the axonal boutons and dendritic spines are highly motile and that a significant fraction has a lifetime of less than a month (Trachtenberg et al., 2002; De Paola et al., 2006). Spine morphology appears to be a reliable predictor of the spine lifetime because transient spines have a smaller spine volume than persistent spines (Holtmaat et al., 2005). Furthermore, while the length of persistent spines remains relatively constant throughout their existence, a high degree of fluctuation could be observed in the length of transient spines. The newly formed spines can rapidly integrate into functional circuits because glutamate receptor currents can be detected in nascent spines that grow either *de novo* in response to glutamate uncaging stimulation or spontaneously in organotypic hippocampal slice cultures (Zito et al., 2009; Kwon and Sabatini, 2011). *Post-hoc* EM reconstruction of spines approximately 1.5 h after their formation shows typical PSD hallmarks, indicating that spontaneously grown spines can acquire both anatomical and functional maturity within an hour or two after their formation (Zito et al., 2009). In contrast to the spines that grow spontaneously, nascent spines that form as a result of high-frequency theta-burst stimulation in hippocampal slices seem to acquire a PSD a day after their formation (Nagerl et al., 2007). A retrospective EM study performed in spines whose age was determined by *in vivo* two-photon imaging also indicates that synapse formation is a protracted process and that dendritic spines acquire synaptic contacts only several days after their formation (Knott et al., 2006). Presumably, the extent of delay between spinogenesis and the synaptogenesis depends on the sample preparation and the stimulation protocol used to generate new spines. It will be interesting to examine if *de novo* spines formed by glutamate uncaging harbor a PSD soon after spinogenesis. Although *de novo* spine growth can be reliably induced in different preparations in response to glutamate uncaging stimulus (Kwon and Sabatini, 2011; Hamilton et al., 2012, 2017; Kozorovitskiy et al., 2012; Hill and Zito, 2013), the ultrastructural features of these spines remain unknown as no *post hoc* EM reconstructions have yet been performed.

Retrospective EM has also opened a door for the examination of the structure function relationship in a synapse, as exemplified by correlated changes in the synaptic ultrastructure in response to neuronal activity. Two-photon glutamate uncaging stimulation, which mimics the spatio-temporal pattern of neuronal activity *in vivo*, has shown that dendritic spines increase their volume instantaneously upon receiving the glutamate stimulus and that this structural change is long–lasting and persistent (Matsuzaki et al., 2004; Oh et al., 2015). Excitatory synaptic strength is proportional to the spine head volume; therefore, the increase in head volume may be necessary to support the enduring enhancement of synaptic transmission during long–term potentiation (LTP). Concomitant with the spine head remodeling, the PSD also changed its shape from the macular type to the perforated and segmented types (Sun et al., 2019). Furthermore, the PSD area also increased in response to LTP stimulation (Sun et al., 2019) and this may be necessary to recruit more AMPA-type receptors to the synapses. However, for at least 30 min after LTP stimulation, PSD size did not seem to increase proportionally with head volume (Bosch et al., 2014; Sun et al., 2019). The increase in PSD area was in fact less than expected based on the increase in the head volume. The slower rate of PSD area increase probably indicates that recruitment of the protein scaffolds in the PSD is slower than the plasma membrane recruitment on the spine surface.

Correlative light EM (CLEM) studies have been used to study how synaptic weights vary along the dendritic arbor of a neuron. The electrical properties of dendrites vary as a function of the distance from the soma; therefore, obtaining a precise morphological description of dendritic spines in reference to their position in the dendritic tree is of particular importance. Ideally, one would need to perform a complete reconstruction of several neurons to reveal both the intra and inter-neuronal variability of spine morphology along the dendritic arbors. However, because of limited imaging volume in the lateral plane, it is only possible to perform a partial reconstruction of dendrites with the SBF-SEM and FIB-SEM methods. Even when whole neuron reconstruction is performed by expending several years of work using ATUM-SEM (Kasthuri et al., 2015), the manual segmentation and quantitative analysis of spine morphology of thousands of spines from each neuron is not yet realistic. Our current insight on the distribution of synaptic strength along a dendrite is derived from the complete reconstruction of a limited number of dendritic branches using serial section TEM. By complete reconstruction of three different dendritic branch segments of CA1 apical oblique dendrites (Katz et al., 2009), it was shown that spine density decreases as one moves from the branch origin to the branch ends. Our recent study (Parajuli et al., 2020a), in which we used dendritic diameter as a rough readout of the distance of a dendritic segment from its branch point, also confirmed and extended the initial finding by Katz and colleagues to the cortex and cerebellum. This type of organized structural placement of dendritic spines may be functionally relevant because a high number of synaptic inputs are necessary to cause a significant depolarization in the large-diameter dendrites near branch points.

To examine how the morphological properties of dendritic spines vary with the distance from the soma in hippocampal or cortical neurons, it is customary to image neuropil volume at a couple of different distances from the pyramidal cell layer. However, a given sampled neuropil volume contains spines from the heterogeneous population of cells that can differ in terms of physiological and morphological properties. Although this problem can be largely overcome by retrospective EM reconstruction of Golgi-impregnated gold-toned neurons (Arellano et al., 2007), a study of this nature is limited in the number of neurons that can be analyzed. Volume imaging must be performed at multiple locations from each neuron; therefore, it is extremely labor-demanding to perform this sort of study in several dozen neurons. Nevertheless, a meticulous study by Arellano et al. (2007) convincingly demonstrated that the morphological features of dendritic spines, such as head volume, spine volume, PSD area, neck length, and neck diameter, do not vary with distance from the soma in layer 2/3 pyramidal cells of the mouse visual cortex.

On a technical note, DAB (3,3’-diaminobenzidine) immunoperoxidase staining is often performed to facilitate *post hoc* EM identification of a dendritic segment of interest that was imaged by two-photon microscopy. However, the diffusible immunoperoxidase reaction end-product not only deteriorates the ultrastructure but also obscures the identification of PSD and the intracellular organelles of a dendritic spine. Thus, instead of using an immunohistochemical method to label neurons of interest, an alternative approach, such as near-infrared branding (NIRB), can be applied for retrospective volume EM studies. In this method, the laser fiducial marks generated around the neurons of interest are visible to both two-photon microscopy and EM, making it particularly useful for correlative light EM studies (Bishop et al., 2011; Maco et al., 2013; Takahashi-Nakazato et al., 2019).

## Technical Considerations and Limitations of Volume EM Studies

Although three-dimensional reconstruction is visually appealing and provides a complete picture of neuronal morphology, it is not completely free of technical limitations. First and foremost, it is necessary to bear in mind that only postmortem tissue that has been subjected to a fixation procedure can be visualized by EM. Therefore, because of fixation artifacts, the EM technique may not necessarily reveal the true structure of dendritic spines in the living state. For example, cup-shaped spines are frequently observed in live preparations (Roelandse et al., 2003; Nagerl et al., 2008). However, very few cup-shaped spines are observed in EM reconstructed images. The ultrastructure of neurons also varies depending on the choice of fixative. Compared with rapid cryofixation of tissue, aldehyde-based chemical fixatives greatly reduce the space occupied by the extracellular matrix and increase the spine neck width (Korogod et al., 2015; Tamada et al., 2020). Therefore, great care should be taken in interpreting EM data and special caution should be applied when comparing data obtained from using different fixation conditions.

EM techniques are still limited to the reconstruction of local neuronal networks in a small volume. Although an extensive neuropil volume can be imaged in the axial direction, current EM techniques have a limited field of view in the lateral direction. However, even in a limited volume, a considerable effort is needed for the manual annotation of data sets such that the effort needed for segmentation far exceeds the effort for imaging. This becomes an issue with connectomics studies (Mishchenko et al., 2010; Kasthuri et al., 2015; Motta et al., 2019) where dense saturated reconstructions of neuropil are necessary. Thus, there is a critical limitation in the number of animals that can be examined for volume EM studies. This raises a genuine concern about inter-animal variability and, therefore, the reproducibility of data sets. To surmount these limitations, several automated segmentation tools have recently been developed that use machine learning approaches (Arganda-Carreras et al., 2015; Berger et al., 2018; Lee et al., 2019; Urakubo et al., 2019) and the continuous refinement of segmentation tools aims to make their accuracy similar to that of a human annotator. With the future development of imaging and analysis tools, it will be possible to substantially increase the throughput of three-dimensional studies.

This review particularly focuses on the three-dimensional structure of excitatory synapses. Inhibitory synapses are as important as excitatory synapses for maintaining proper excitation-inhibition balance in the neuronal network. Compared with excitatory synapses, fewer volume EM studies have studied inhibitory synapses (Merchan-Perez et al., 2009; Santuy et al., 2018, 2020; Kwon et al., 2019). Inhibitory synapses are mainly made on the dendritic shafts and they do not have prominent postsynaptic thickenings. As such, identification of symmetric, inhibitory synapses is somewhat difficult compared with the identification of asymmetric synapses of dendritic spines. Further technical refinement, such as devising appropriate sample preparation protocols that permit distinct visualization of symmetric inhibitory synapses, and improvements in instrumentation and engineering to increase the resolution limit of SEM methods, are necessary to undertake large-scale three-dimensional ultrastructural studies of inhibitory synapses.

## Author Contributions

LKP and MK drafted, edited and finalized the manuscript, and designed/modified figures. All authors contributed to the article and approved the submitted version.

## Conflict of Interest

The authors declare that the research was conducted in the absence of any commercial or financial relationships that could be construed as a potential conflict of interest.

## References

[B1] Arellano J. I. Benavides-Piccione R. DeFelipe J. Yuste R. (2007). Ultrastructure of dendritic spines: correlation between synaptic and spine morphologies. Front. Neurosci. 1, 131–143. 10.3389/neuro.01.1.1.010.2007 18982124 PMC2518053

[B2] Arganda-Carreras I. Turaga S. C. Berger D. R. Ciresan D. Giusti A. Gambardella L. M.. (2015). Crowdsourcing the creation of image segmentation algorithms for connectomics. Front. Neuroanat. 9:142. 10.3389/fnana.2015.00142 26594156 PMC4633678

[B3] Aziz W. Wang W. Kesaf S. Mohamed A. A. Fukazawa Y. Shigemoto R. (2014). Distinct kinetics of synaptic structural plasticity, memory formation and memory decay in massed and spaced learning. Proc. Natl. Acad. Sci. U S A 111, E194–E202. 10.1073/pnas.1303317110 24367076 PMC3890840

[B4] Bartol T. M. Bromer C. Kinney J. Chirillo M. A. Bourne J. N. Harris K. M.. (2015). Nanoconnectomic upper bound on the variability of synaptic plasticity. eLife 4:e10778. 10.7554/eLife.10778 26618907 PMC4737657

[B5] Berger D. R. Seung H. S. Lichtman J. W. (2018). VAST (Volume Annotation and Segmentation Tool): efficient manual and semi-automatic labeling of large 3D image stacks. Front. Neural. Circuits 12:88. 10.3389/fncir.2018.00088 30386216 PMC6198149

[B6] Bishop D. Nikic I. Brinkoetter M. Knecht S. Potz S. Kerschensteiner M.. (2011). Near-infrared branding efficiently correlates light and electron microscopy. Nat. Methods 8, 568–570. 10.1038/nmeth.1622 21642966

[B7] Bloss E. B. Cembrowski M. S. Karsh B. Colonell J. Fetter R. D. Spruston N. (2018). Single excitatory axons form clustered synapses onto CA1 pyramidal cell dendrites. Nat. Neurosci. 21, 353–363. 10.1038/s41593-018-0084-6 29459763

[B8] Bock D. D. Lee W. C. Kerlin A. M. Andermann M. L. Hood G. Wetzel A. W.. (2011). Network anatomy and *in vivo* physiology of visual cortical neurons. Nature 471, 177–182. 10.1038/nature09802 21390124 PMC3095821

[B9] Bosch C. Martinez A. Masachs N. Teixeira C.M. Fernaud I. Ulloa F.. (2015). FIB/SEM technology and high-throughput 3D reconstruction of dendritic spines and synapses in GFP-labeled adult-generated neurons. Front. Neuroanat. 9:60. 10.3389/fnana.2015.00060 26052271 PMC4440362

[B10] Bosch M. Castro J. Saneyoshi T. Matsuno H. Sur M. Hayashi Y. (2014). Structural and molecular remodeling of dendritic spine substructures during long-term potentiation. Neuron 82, 444–459. 10.1016/j.neuron.2014.03.021 24742465 PMC4281348

[B11] Bourne J. N. Harris K. M. (2008). Balancing structure and function at hippocampal dendritic spines. Annu. Rev. Neurosci. 31, 47–67. 10.1146/annurev.neuro.31.060407.125646 18284372 PMC2561948

[B12] Briggman K. L. Helmstaedter M. Denk W. (2011). Wiring specificity in the direction-selectivity circuit of the retina. Nature 471, 183–188. 10.1038/nature09818 21390125

[B13] Cali C. Wawrzyniak M. Becker C. Maco B. Cantoni M. Jorstad A.. (2018). The effects of aging on neuropil structure in mouse somatosensory cortex-A 3D electron microscopy analysis of layer 1. PLoS One 13:e0198131. 10.1371/journal.pone.0198131 29966021 PMC6028106

[B14] Chereau R. Tonnesen J. Nagerl U. V. (2015). STED microscopy for nanoscale imaging in living brain slices. Methods 88, 57–66. 10.1016/j.ymeth.2015.06.006 26070997

[B15] Chicurel M. E. Harris K. M. (1992). Three-dimensional analysis of the structure and composition of CA3 branched dendritic spines and their synaptic relationships with mossy fiber boutons in the rat hippocampus. J. Comp. Neurol. 325, 169–182. 10.1002/cne.903250204 1460112

[B16] De Paola V. Holtmaat A. Knott G. Song S. Wilbrecht L. Caroni P.. (2006). Cell type-specific structural plasticity of axonal branches and boutons in the adult neocortex. Neuron 49, 861–875. 10.1016/j.neuron.2006.02.017 16543134

[B17] Denk W. Horstmann H. (2004). Serial block-face scanning electron microscopy to reconstruct three-dimensional tissue nanostructure. PLoS Biol. 2:e329. 10.1371/journal.pbio.0020329 15514700 PMC524270

[B18] Dominguez-Alvaro M. Montero-Crespo M. Blazquez-Llorca L. DeFelipe J. Alonso-Nanclares L. (2019). 3D electron microscopy study of synaptic organization of the normal human transentorhinal cortex and its possible alterations in Alzheimer’s disease. eNeuro 6:ENEURO.0140-19.2019. 10.1523/ENEURO.0140-19.2019 31217195 PMC6620390

[B19] Dominguez-Alvaro M. Montero-Crespo M. Blazquez-Llorca L. Insausti R. DeFelipe J. Alonso-Nanclares L. (2018). Three-dimensional analysis of synapses in the transentorhinal cortex of Alzheimer’s disease patients. Acta Neuropathol. Commun. 6:20. 10.1186/s40478-018-0520-6 29499755 PMC5834884

[B20] Fang T. Lu X. Berger D. Gmeiner C. Cho J. Schalek R.. (2018). Nanobody immunostaining for correlated light and electron microscopy with preservation of ultrastructure. Nat. Methods 15, 1029–1032. 10.1038/s41592-018-0177-x 30397326 PMC6405223

[B21] Frotscher M. Studer D. Graber W. Chai X. Nestel S. Zhao S. (2014). Fine structure of synapses on dendritic spines. Front. Neuroanat. 8:94. 10.3389/fnana.2014.00094 25249945 PMC4158982

[B22] Hamilton A. M. Lambert J. T. Parajuli L. K. Vivas O. Park D. K. Stein I. S.. (2017). A dual role for the RhoGEF Ephexin5 in regulation of dendritic spine outgrowth. Mol. Cell Neurosci. 80, 66–74. 10.1016/j.mcn.2017.02.001 28185854 PMC5526822

[B23] Hamilton A. M. Oh W. C. Vega-Ramirez H. Stein I. S. Hell J. W. Patrick G. N.. (2012). Activity-dependent growth of new dendritic spines is regulated by the proteasome. Neuron 74, 1023–1030. 10.1016/j.neuron.2012.04.031 22726833 PMC3500563

[B24] Hamos J. E. Van Horn S. C. Raczkowski D. Sherman S. M. (1987). Synaptic circuits involving an individual retinogeniculate axon in the cat. J. Comp. Neurol. 259, 165–192. 10.1002/cne.902590202 3584556

[B25] Harris K. M. Stevens J. K. (1988). Dendritic spines of rat cerebellar Purkinje cells: serial electron microscopy with reference to their biophysical characteristics. J. Neurosci. 8, 4455–4469. 10.1523/JNEUROSCI.08-12-04455.1988 3199186 PMC6569567

[B26] Harris K. M. Stevens J. K. (1989). Dendritic spines of CA1 pyramidal cells in the rat hippocampus: serial electron microscopy with reference to their biophysical characteristics. J. Neurosci. 9, 2982–2997. 10.1523/JNEUROSCI.09-08-02982.1989 2769375 PMC6569708

[B27] Harris K. M. Weinberg R. J. (2012). Ultrastructure of synapses in the mammalian brain. Cold. Spring. Harbor. Perspect. Biol. 4:a005587. 10.1101/cshperspect.a005587 22357909 PMC3331701

[B28] Harris K. M. Jensen F. E. Tsao B. (1992). Three-dimensional structure of dendritic spines and synapses in rat hippocampus (CA1) at postnatal day 15 and adult ages: implications for the maturation of synaptic physiology and long-term potentiation. J. Neurosci. 12, 2685–2705. 10.1523/JNEUROSCI.12-07-02685.1992 1613552 PMC6575840

[B29] Harris K. M. Perry E. Bourne J. Feinberg M. Ostroff L. Hurlburt J. (2006). Uniform serial sectioning for transmission electron microscopy. J. Neurosci. 26, 12101–12103. 10.1523/JNEUROSCI.3994-06.2006 17122034 PMC6675417

[B30] Hill T. C. Zito K. (2013). LTP-induced long-term stabilization of individual nascent dendritic spines. J. Neurosci. 33, 678–686. 10.1523/JNEUROSCI.1404-12.2013 23303946 PMC6704923

[B31] Holtmaat A. J. Trachtenberg J. T. Wilbrecht L. Shepherd G. M. Zhang X. Knott G. W.. (2005). Transient and persistent dendritic spines in the neocortex *in vivo*. Neuron 45, 279–291. 10.1016/j.neuron.2005.01.003 15664179

[B32] Ichikawa R. Hashimoto K. Miyazaki T. Uchigashima M. Yamasaki M. Aiba A.. (2016). Territories of heterologous inputs onto Purkinje cell dendrites are segregated by mGluR1-dependent parallel fiber synapse elimination. Proc. Natl. Acad. Sci. U S A 113, 2282–2287. 10.1073/pnas.1511513113 26858447 PMC4776453

[B33] Ichikawa R. Miyazaki T. Kano M. Hashikawa T. Tatsumi H. Sakimura K.. (2002). Distal extension of climbing fiber territory and multiple innervation caused by aberrant wiring to adjacent spiny branchlets in cerebellar Purkinje cells lacking glutamate receptor δ2. J. Neurosci. 22, 8487–8503. 10.1523/JNEUROSCI.22-19-08487.2002 12351723 PMC6757771

[B34] Jawaid S. Kidd G. J. Wang J. Swetlik C. Dutta R. Trapp B. D. (2018). Alterations in CA1 hippocampal synapses in a mouse model of fragile X syndrome. Glia 66, 789–800. 10.1002/glia.23284 29274095 PMC5812820

[B35] Jiang Z. Jin X. Li Y. Liu S. Liu X. M. Wang Y. Y.. (2020). Genetically encoded tags for direct synthesis of EM-visible gold nanoparticles in cells. Nat. Methods 17, 937–946. 10.1038/s41592-020-0911-z 32778831

[B36] Kashiwagi Y. Higashi T. Obashi K. Sato Y. Komiyama N. H. Grant S. G. N.. (2019). Computational geometry analysis of dendritic spines by structured illumination microscopy. Nat. Commun. 10:1285. 10.1038/s41467-019-09337-0 30894537 PMC6427002

[B37] Kasthuri N. Hayworth K. J. Berger D. R. Schalek R. L. Conchello J. A. Knowles-Barley S.. (2015). Saturated reconstruction of a volume of neocortex. Cell 162, 648–661. 10.1016/j.cell.2015.06.054 26232230

[B38] Katz Y. Menon V. Nicholson D. A. Geinisman Y. Kath W. L. Spruston N. (2009). Synapse distribution suggests a two-stage model of dendritic integration in CA1 pyramidal neurons. Neuron 63, 171–177. 10.1016/j.neuron.2009.06.023 19640476 PMC2921807

[B39] Knott G. Marchman H. Wall D. Lich B. (2008). Serial section scanning electron microscopy of adult brain tissue using focused ion beam milling. J. Neurosci. 28, 2959–2964. 10.1523/JNEUROSCI.3189-07.2008 18353998 PMC6670719

[B40] Knott G. W. Holtmaat A. Wilbrecht L. Welker E. Svoboda K. (2006). Spine growth precedes synapse formation in the adult neocortex *in vivo*. Nat. Neurosci. 9, 1117–1124. 10.1038/nn1747 16892056

[B41] Koike M. (2018). Denshi kenbikyōsan jigen rittai sai kochiku ni yoru soshiki saibō no kansatsu no yūyō-sei to kongo no kadai [Usefulness and future perspectives of volume electron microscopy for 3D analysis of cells and tissues]. LiSA 25, 79–89.

[B42] Korogod N. Petersen C. C. Knott G. W. (2015). Ultrastructural analysis of adult mouse neocortex comparing aldehyde perfusion with cryo fixation. eLife 4:e05793. 10.7554/eLife.05793 26259873 PMC4530226

[B43] Kozorovitskiy Y. Saunders A. Johnson C. A. Lowell B. B. Sabatini B. L. (2012). Recurrent network activity drives striatal synaptogenesis. Nature 485, 646–650. 10.1038/nature11052 22660328 PMC3367801

[B44] Kubota Y. Hatada S. Kondo S. Karube F. Kawaguchi Y. (2007). Neocortical inhibitory terminals innervate dendritic spines targeted by thalamocortical afferents. J. Neurosci. 27, 1139–1150. 10.1523/JNEUROSCI.3846-06.2007 17267569 PMC6673192

[B45] Kulik Y. D. Watson D. J. Cao G. Kuwajima M. Harris K. M. (2019). Structural plasticity of dendritic secretory compartments during LTP-induced synaptogenesis. eLife 8:e46356. 10.7554/eLife.46356 31433297 PMC6728136

[B46] Kwon H. B. Sabatini B. L. (2011). Glutamate induces *de novo* growth of functional spines in developing cortex. Nature 474, 100–104. 10.7554/eLife.46356 21552280 PMC3107907

[B47] Kwon T. Merchan-Perez A. Rial Verde E. M. Rodriguez J. R. DeFelipe J. Yuste R. (2019). Ultrastructural, molecular and functional mapping of GABAergic synapses on dendritic spines and shafts of neocortical pyramidal neurons. Cereb. Cortex 29, 2771–2781. 10.1093/cercor/bhy143 30113619 PMC6611501

[B48] Lam S. S. Martell J. D. Kamer K. J. Deerinck T. J. Ellisman M. H. Mootha V. K.. (2015). Directed evolution of APEX2 for electron microscopy and proximity labeling. Nat. Methods 12, 51–54. 10.1038/nmeth.3179 25419960 PMC4296904

[B49] Lee K. Turner N. Macrina T. Wu J. Lu R. Seung H. S. (2019). Convolutional nets for reconstructing neural circuits from brain images acquired by serial section electron microscopy. Curr. Opin. Neurobiol. 55, 188–198. 10.1016/j.conb.2019.04.001 31071619 PMC6559369

[B50] Lee W. C. Bonin V. Reed M. Graham B. J. Hood G. Glattfelder K.. (2016). Anatomy and function of an excitatory network in the visual cortex. Nature 532, 370–374. 10.1038/nature17192 27018655 PMC4844839

[B51] Lenn N. J. (1976). Synapses in the interpeduncular nucleus: electron microscopy of normal and habenula lesioned rats. J. Comp. Neurol. 166, 77–99. 10.1002/cne.901660106 1262550

[B52] Maco B. Holtmaat A. Cantoni M. Kreshuk A. Straehle C. N. Hamprecht F. A.. (2013). Correlative *in vivo* 2 photon and focused ion beam scanning electron microscopy of cortical neurons. PLoS One 8:e57405. 10.1371/journal.pone.0057405 23468982 PMC3585404

[B53] Masugi-Tokita M. Shigemoto R. (2007). High-resolution quantitative visualization of glutamate and GABA receptors at central synapses. Curr. Opin. Neurobiol. 17, 387–393. 10.1016/j.conb.2007.04.012 17499496

[B55] Matsuzaki M. Honkura N. Ellis-Davies G. C. Kasai H. (2004). Structural basis of long-term potentiation in single dendritic spines. Nature 429, 761–766. 10.1038/nature02617 15190253 PMC4158816

[B56] Medvedev N. I. Dallerac G. Popov V. I. Rodriguez Arellano J. J. Davies H. A. Kraev I. V.. (2014). Multiple spine boutons are formed after long-lasting LTP in the awake rat. Brain Struct. Funct. 219, 407–414. 10.1007/s00429-012-0488-0 23224218

[B57] Medvedev N. I. Popov V. I. Dallerac G. Davies H. A. Laroche S. Kraev I. V.. (2010). Alterations in synaptic curvature in the dentate gyrus following induction of long-term potentiation, long-term depression and treatment with the N-methyl-D-aspartate receptor antagonist CPP. Neuroscience 171, 390–397. 10.1016/j.neuroscience.2010.09.014 20849931

[B58] Merchan-Perez A. Rodriguez J. R. Alonso-Nanclares L. Schertel A. DeFelipe J. (2009). Counting synapses using FIB/SEM microscopy: A true revolution for ultrastructural volume reconstruction. Front. Neuroanat. 3:18. 10.3389/neuro.05.018.2009 19949485 PMC2784681

[B59] Mikuni T. Nishiyama J. Sun Y. Kamasawa N. Yasuda R. (2016). High-throughput, high-resolution mapping of protein localization in mammalian brain by *in vivo* genome editing. Cell 165, 1803–1817. 10.1016/j.cell.2016.04.044 27180908 PMC4912470

[B60] Mishchenko Y. Hu T. Spacek J. Mendenhall J. Harris K. M. Chklovskii D. B. (2010). Ultrastructural analysis of hippocampal neuropil from the connectomics perspective. Neuron 67, 1009–1020. 10.1016/j.neuron.2010.08.014 20869597 PMC3215280

[B61] Motta A. Berning M. Boergens K. M. Staffler B. Beining M. Loomba S.. (2019). Dense connectomic reconstruction in layer 4 of the somatosensory cortex. Science 366:eaay3134. 10.1126/science.aay3134 31649140

[B62] Nagerl U. V. Kostinger G. Anderson J. C. Martin K. A. Bonhoeffer T. (2007). Protracted synaptogenesis after activity-dependent spinogenesis in hippocampal neurons. J. Neurosci. 27, 8149–8156. 10.1523/JNEUROSCI.0511-07.2007 17652605 PMC6672732

[B63] Nagerl U. V. Willig K. I. Hein B. Hell S. W. Bonhoeffer T. (2008). Live-cell imaging of dendritic spines by STED microscopy. Proc. Natl. Acad. Sci. U S A 105, 18982–18987. 10.1073/pnas.0810028105 19028874 PMC2585941

[B64] Nakao A. Miyazaki N. Ohira K. Hagihara H. Takagi T. Usuda N.. (2017). Immature morphological properties in subcellular-scale structures in the dentate gyrus of Schnurri-2 knockout mice: a model for schizophrenia and intellectual disability. Mol. Brain 10:60. 10.1186/s13041-017-0339-2 29233179 PMC5727961

[B65] Nicholson D. A. Trana R. Katz Y. Kath W. L. Spruston N. Geinisman Y. (2006). Distance-dependent differences in synapse number and AMPA receptor expression in hippocampal CA1 pyramidal neurons. Neuron 50, 431–442. 10.1016/j.neuron.2006.03.022 16675397

[B66] Oh W. C. Parajuli L. K. Zito K. (2015). Heterosynaptic structural plasticity on local dendritic segments of hippocampal CA1 neurons. Cell Rep. 10, 162–169. 10.1016/j.celrep.2014.12.016 25558061 PMC4294981

[B67] Okabe S. (2020). Recent advances in computational methods for measurement of dendritic spines imaged by light microscopy. Microscopy 69, 196–213. 10.1093/jmicro/dfaa016 32244257

[B68] Ostroff L. E. Fiala J. C. Allwardt B. Harris K. M. (2002). Polyribosomes redistribute from dendritic shafts into spines with enlarged synapses during LTP in developing rat hippocampal slices. Neuron 35, 535–545. 10.1016/s0896-6273(02)00785-7 12165474

[B69] Parajuli L. K. (2018). Technical toolbox for decoding synaptic complexity. Juntendo Med. J. 64, 386–391. 10.14789/jmj.2018.64.jmj18-ln03

[B70] Parajuli L. K. Fukazawa Y. Watanabe M. Shigemoto R. (2010). Subcellular distribution of α1G subunit of T-type calcium channel in the mouse dorsal lateral geniculate nucleus. J. Comp. Neurol. 518, 4362–4374. 10.1002/cne.22461 20853512

[B71] Parajuli L. K. Nakajima C. Kulik A. Matsui K. Schneider T. Shigemoto R.. (2012). Quantitative regional and ultrastructural localization of the Ca(v)2.3 subunit of R-type calcium channel in mouse brain. J. Neurosci. 32, 13555–13567. 10.1523/JNEUROSCI.1142-12.2012 23015445 PMC6621359

[B72] Parajuli L. K. Tanaka S. Okabe S. (2017). Insights into age-old questions of new dendritic spines: from form to function. Brain Res. Bull. 129, 3–11. 10.1016/j.brainresbull.2016.07.014 27491624

[B73] Parajuli L. K. Urakubo H. Takahashi-Nakazato A. Ogelman R. Iwasaki H. Koike M.. (2020a). Geometry and the organizational principle of spine synapses along a dendrite. eNeuro 7:ENEURO.0248-20.2020. 10.1523/ENEURO.0248-20.2020 33109633 PMC7772515

[B74] Parajuli L. K. Wako K. Maruo S. Kakuta S. Koike M. (2020b). Unique synaptic topography of crest-type synapses in the interpeduncular nucleus. Biochem. Biophys. Res. Commun. 530, 130–135. 10.1016/j.bbrc.2020.06.046 32828274

[B75] Parajuli L. K. Wako K. Maruo S. Kakuta S. Taguchi T. Ikuno M.. (2020c). Developmental changes in dendritic spine morphology in the striatum and their alteration in an A53T α-synuclein transgenic mouse model of Parkinson’s disease. eNeuro 7:ENEURO.0072-20.2020. 10.1523/ENEURO.0072-20.2020 32817196 PMC7470930

[B76] Penzes P. Cahill M. E. Jones K. A. VanLeeuwen J. E. Woolfrey K. M. (2011). Dendritic spine pathology in neuropsychiatric disorders. Nat. Neurosci. 14, 285–293. 10.1038/nn.2741 21346746 PMC3530413

[B77] Popov V. I. Kleschevnikov A. M. Klimenko O. A. Stewart M. G. Belichenko P. V. (2011). Three-dimensional synaptic ultrastructure in the dentate gyrus and hippocampal area CA3 in the Ts65Dn mouse model of Down syndrome. J. Comp. Neurol. 519, 1338–1354. 10.1002/cne.22573 21452200

[B78] Roelandse M. Welman A. Wagner U. Hagmann J. Matus A. (2003). Focal motility determines the geometry of dendritic spines. Neuroscience 121, 39–49. 10.1016/s0306-4522(03)00405-6 12946698

[B79] Sanculi D. Pannoni K. E. Bushong E. A. Crump M. Sung M. Popat V.. (2020). Toric spines at a site of learning. eNeuro 7:ENEURO.0197-19.2019. 10.1523/ENEURO.0197-19.2019 31822521 PMC6944481

[B80] Santuy A. Rodriguez J. R. DeFelipe J. Merchan-Perez A. (2018). Volume electron microscopy of the distribution of synapses in the neuropil of the juvenile rat somatosensory cortex. Brain Struct. Funct. 223, 77–90. 10.1007/s00429-017-1470-7 28721455 PMC5772167

[B81] Santuy A. Tomas-Roca L. Rodriguez J. R. Gonzalez-Soriano J. Zhu F. Qiu Z.. (2020). Estimation of the number of synapses in the hippocampus and brain-wide by volume electron microscopy and genetic labeling. Sci. Rep. 10:14014. 10.1038/s41598-020-70859-5 32814795 PMC7438319

[B82] Sato Y. Okabe S. (2019). Nano-scale analysis of synapse morphology in an autism mouse model with 15q11–13 copy number variation using focused ion beam milling and scanning electron microscopy. Microscopy 68, 122–132. 10.1093/jmicro/dfy128 30371805

[B83] Shu X. Lev-Ram V. Deerinck T. J. Qi Y. Ramko E. B. Davidson M. W.. (2011). A genetically encoded tag for correlated light and electron microscopy of intact cells, tissues and organisms. PLoS Biol. 9:e1001041. 10.1371/journal.pbio.1001041 21483721 PMC3071375

[B84] Stewart M. Popov V. Medvedev N. Gabbott P. Corbett N. Kraev I.. (2010). Dendritic spine and synapse morphological alterations induced by a neural cell adhesion molecule mimetic. Adv. Exp. Med. Biol. 663, 373–383. 10.1007/978-1-4419-1170-4_23 20017034

[B85] Stewart M. G. Medvedev N. I. Popov V. I. Schoepfer R. Davies H. A. Murphy K.. (2005). Chemically induced long-term potentiation increases the number of perforated and complex postsynaptic densities but does not alter dendritic spine volume in CA1 of adult mouse hippocampal slices. Eur. J. Neurosci. 21, 3368–3378. 10.1111/j.1460-9568.2005.04174.x 16026474

[B86] Sun Y. Smirnov M. Kamasawa N. Yasuda R. (2019). Rapid ultrastructural changes of PSD and extrasynaptic axon-spine interface membrane during LTP induced in single dendritic spine. bioRxiv [Preprint]. 10.1101/840629

[B87] Takahashi-Nakazato A. Parajuli L. K. Iwasaki H. Tanaka S. Okabe S. (2019). Ultrastructural observation of glutamatergic synapses by focused ion beam scanning electron microscopy (FIB/SEM). Methods Mol. Biol. 1941, 17–27. 10.1007/978-1-4939-9077-1_2 30707424

[B88] Tamada H. Blanc J. Korogod N. Petersen C. Knott G. (2020). Ultrastructural comparison of dendritic spine morphology preserved with cryo and chemical fixation. eLife 9:e56384. 10.7554/eLife.56384 33274717 PMC7748412

[B89] Trachtenberg J. T. Chen B. E. Knott G. W. Feng G. Sanes J. R. Welker E.. (2002). Long-term *in vivo* imaging of experience-dependent synaptic plasticity in adult cortex. Nature 420, 788–794. 10.1038/nature01273 12490942

[B90] Urakubo H. Bullmann T. Kubota Y. Oba S. Ishii S. (2019). UNI-EM: An environment for deep neural network-based automated segmentation of neuronal electron microscopic images. Sci. Rep. 9:19413. 10.1038/s41598-019-55431-0 31857624 PMC6923391

[B91] Van de Plas P. Leunissen J. L. (1993). Ultrasmall gold probes: characteristics and use in immuno(cyto)chemical studies. Methods Cell Biol. 37, 241–257. 10.1016/s0091-679x(08)60253-8 8255246

[B92] White J. G. Southgate E. Thomson J. N. Brenner S. (1986). The structure of the nervous system of the nematode Caenorhabditis elegans. Philos. Trans. R Soc. Lond B Biol. Sci. 314, 1–340. 10.1098/rstb.1986.0056 22462104

[B93] Wilke S. A. Antonios J. K. Bushong E. A. Badkoobehi A. Malek E. Hwang M.. (2013). Deconstructing complexity: serial block-face electron microscopic analysis of the hippocampal mossy fiber synapse. J. Neurosci. 33, 507–522. 10.1523/JNEUROSCI.1600-12.2013 23303931 PMC3756657

[B94] Wilson C. J. Groves P. M. Kitai S. T. Linder J. C. (1983). Three-dimensional structure of dendritic spines in the rat neostriatum. J. Neurosci. 3, 383–388. 10.1523/JNEUROSCI.03-02-00383.1983 6822869 PMC6564480

[B95] Witcher M. R. Park Y. D. Lee M. R. Sharma S. Harris K. M. Kirov S. A. (2010). Three-dimensional relationships between perisynaptic astroglia and human hippocampal synapses. Glia 58, 572–587. 10.1002/glia.20946 19908288 PMC2845925

[B96] Zito K. Scheuss V. Knott G. Hill T. Svoboda K. (2009). Rapid functional maturation of nascent dendritic spines. Neuron 61, 247–258. 10.1016/j.neuron.2008.10.054 19186167 PMC2800307

